# A novel surgical technique for repairing duodenal and bile duct perforations following endoscopic retrograde cholangiopancreatography

**DOI:** 10.1093/jscr/rjaf050

**Published:** 2025-02-11

**Authors:** Hana Abdirahman, Omar Barakat, Alexis Nichols, Oluwatobi Soares, Jared Mortus, Vivi Chen

**Affiliations:** Department of Surgery, Division of Surgical Oncology and Hepatobiliary and Pancreas Surgery, Baylor College of Medicine, 7200 Cambridge Street, Houston, TX 77030-3411, United States; Department of Surgery, Division of Surgical Oncology and Hepatobiliary and Pancreas Surgery, Baylor College of Medicine, 7200 Cambridge Street, Houston, TX 77030-3411, United States; Department of Surgery, Division of Surgical Oncology and Hepatobiliary and Pancreas Surgery, Baylor College of Medicine, 7200 Cambridge Street, Houston, TX 77030-3411, United States; Department of Surgery, Division of Surgical Oncology and Hepatobiliary and Pancreas Surgery, Baylor College of Medicine, 7200 Cambridge Street, Houston, TX 77030-3411, United States; Department of Surgery, Division of Surgical Oncology and Hepatobiliary and Pancreas Surgery, Baylor College of Medicine, 7200 Cambridge Street, Houston, TX 77030-3411, United States; Department of Surgery, Division of Surgical Oncology and Hepatobiliary and Pancreas Surgery, Baylor College of Medicine, 7200 Cambridge Street, Houston, TX 77030-3411, United States

**Keywords:** case report, duodenum, bile duct injury, duodenal perforation, ERCP, Roux-en-Y

## Abstract

Duodenal perforation (DP), though rare, is a severe complication of Endoscopic retrograde cholangiopancreatography (ERCP) with high mortality rates. This report introduces a novel surgical approach for repairing a complex combined bile duct and duodenal perforation. A 37-year-old male with recurrent pyloric stenosis and choledocholithiasis, previously treated with multiple procedures, presented with gastric outlet and bile duct obstruction. Following a complex ERCP, he developed a large combined duodenal and bile duct perforation requiring urgent surgical intervention. A 40% circumferential duodenal perforation combined with bile duct perforation was repaired using a novel approach: a vascularized isolated distal gastric pouch was created and anastomosed to the duodenal and bile duct defects. A Roux-en-Y gastrojejunostomy was performed, and the patient recovered in stable condition. When traditional reconstruction is not feasible for DP, an isolated, vascularized distal gastric pouch offers a less invasive alternative and reduces the risk of morbidity.

## Introduction

Endoscopic retrograde cholangiopancreatography (ERCP) has become an essential tool for diagnosing and treating pancreatic and biliary disorders. However, its use is associated with complications such as pancreatitis, bleeding, and cholangitis. Duodenal perforation (DP), though rare, is a severe complication of ERCP with an incidence of approximately 1% and a high mortality rate ranging from 4.5% to 33% [1–3]. Traditional management strategies often result in increased morbidity and prolonged recovery, particularly in cases of large, non-contained perforations. Here, we present a novel surgical approach for repairing a challenging combined bile duct perforation and DP that could not be addressed with standard techniques. This innovative method highlights the importance of advancing treatment strategies for complex ERCP complications and offers a promising solution for managing this life-threatening condition.

## Case report

**Figure 1:**
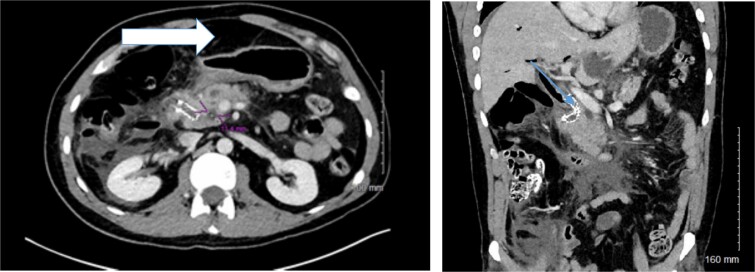
Preoperative and intraoperative findings of duodenal perforation and reconstruction. Preoperative CT- scan of the abdomen delineating the amount of free intra-peritoneal air (arrow) and the location of the biliary metallic stent (arrow).

A 37-year-old male with a history of recurrent pyloric stenosis and choledocholithiasis, previously treated with laparoscopic Heineke-Mikulicz pyloroplasty, cholecystectomy, multiple pyloric dilations, and ERCP, presented with recurrent gastric outlet obstruction and bile duct obstruction. He underwent a complex ERCP procedure to extract a common bile duct stone and place a biliary stent. However, due to a deformed duodenum and complex biliary anatomy, the procedure was complicated by a large combined duodenal and bile duct perforation confirmed on CT-scan imaging (Fig. 1) 4 days later and necessitated urgent surgical intervention.

### Surgical technique

**Figure 2:**
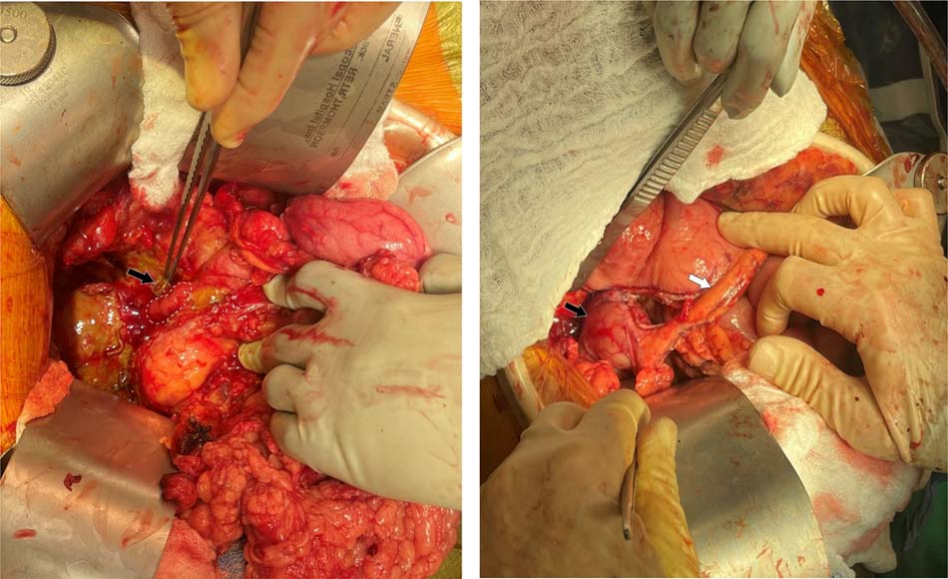
Intraoperative findings and final reconstruction using vascularized isolated distal gastric pouch. (A) Intraoperative findings of the duodenal perforation (dotted circle) and the metallic stent protruding through the bile duct (arrow). (B) Final reconstruction using vascularized isolated distal gastric pouch (black arrow) maintaining the gastroepiploic vascular pedicle to the pouch (arrow).

The patient underwent an exploratory laparotomy via an upper midline incision. Dense adhesions were released, and large peri-hepatic biloma was drained. A 40% circumferential perforation of the proximal second portion of the duodenum was identified with a metallic stent protruding from the supra-pancreatic common bile duct (CBD) into the perforation (Fig. 2). The stent was removed, and intra-operative choledochoscopy confirmed the absence of any residual stones.

The first portion of the duodenum was divided above the perforation using a stapling device following complete mobilization of the duodenum. A double gastric and duodenal anastomosis was planned to utilize the proximal jejunum. The proximal jejunum was divided ~ 40 cm from the ligament of Treitz. However, attempts to mobilize the distal jejunal loop to reach the duodenal and bile duct defect was unsuccessful due to severe inflammation, and short mesentery.

**Figure 3:**
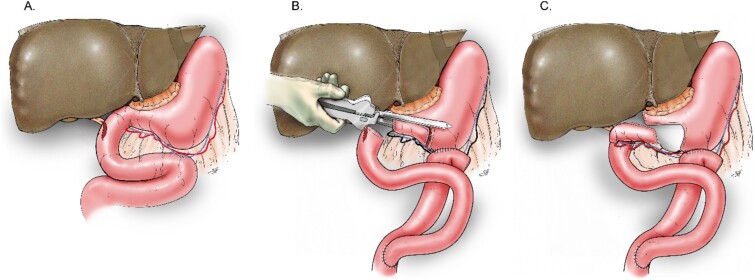
Schematic delineating the reconstruction technique for the vascularized, isolated gastric pouch. (A) Depiction of the site of the duodenal and bile duct injury. (B) Creation of the distal gastric pouch. (C) Final anastomoses of the pouch to the duodenal and bile duct defect.

To address this, a 6 cm isolated vascularized distal gastric pouch was created by longitudinally dividing the distal stomach at the staple line, then isolating it from the proximal stomach using a stapling device, while preserving the gastroepiploic blood supply to the pouch. The pouch was anastomosed to the duodenal and CBD defects using interrupted 4–0 PDS sutures in an end-to-side manner. A Roux-en-Y gastrojejunostomy was then constructed in standard fashion (Figs 2 and 3).

Two Jackson-Pratt drains were placed anterior and posterior to the pouch. The patient recovered in stable condition.

### Post-operative course

On post operative day (POD) 2, the drain output was noted to be bilious in nature. An internal/external percutaneous transhepatic biliary drainage catheter (PTBDC) was placed by an interventional radiologist, resulting in complete resolution of the biliary leakage. Following the procedure, serum bilirubin and other liver function test (LFT) values demonstrated a downward trend. Patient was maintained on broad spectrum antibiotics and then tailored according to the intra-operative culture result. The post operative course was also complicated by recurrent right pleural effusion, which was treated successfully with a chest tube. The chest tube was removed following complete resolution of the pleural effusion. The PTBDC was the internalized upon near normalization of the LFTs. Given the patients clinical stability, he was discharged on POD 17 with close outpatient follow-up.

## Discussion

DP after ERCP is a rare but potentially life-threatening complication. As endoscopic techniques and devices advance, the incidence of complex complications may also increase. Management of DP varies based on the severity and type of perforation, with options including conservative, endoscopic, and surgical approaches.

Conservative management is suitable for stable patients with contained perforations. For minor uncontained perforations, endoscopic approaches, and surgical patch repairs, are commonly employed and have demonstrated consistently favorable outcomes [4, 5]. Traumatic DP or cases involving hemodynamically unstable patients may benefit from the triple-tube technique. This involves primary defect repair combined with decompressing tube gastrostomy, duodenostomy, and feeding jejunostomy. This approach has shown significantly better outcomes in terms of both short- and long-term morbidity and mortality compared to omentopexy [4, 6].

For larger DPs with viable surrounding tissue, duodenojejunostomy is a viable surgical option. More extensive repairs or other complex reconstructive procedures are typically reserved for cases involving the pancreaticobiliary system [5, 6]. Pancreaticoduodenectomy, while the most invasive repair option, carries a significant risk of postoperative complications and is generally considered a last resort [7].

In the present case, the size of the duodenal defect precluded the use of a patch or primary repair. Due to significant inflammation and limited small bowel mobility, traditional duodenojejunostomy and other reconstructive surgeries were not viable options. Instead of opting for pancreaticoduodenectomy, a vascularized isolated distal gastric pouch was created and anastomosed to both the duodenal and biliary defects. Multiple drains were placed prophylactically to manage potential postoperative leakage. Additionally, bile diversion via percutaneous transhepatic biliary drainage catheter was employed, resulting in a favorable outcome.

## Conclusion

Many factors should be considered when determining the appropriate surgical management of DP including size, tissue viability, location, and the patient's hemodynamic status. When primary repair and traditional reconstruction are not viable options, employing an isolated, vascularized distal gastric pouch may be a suitable alternative, potentially reducing risk of morbidity associated with more extensive surgical procedures.

## References

[1] Enns R, Eloubeidi MA, Mergener K, et al. ERCP-related perforations: risk factors and management. Endoscopy 2002;34:293–8. 10.1055/s-2002-23650. 11932784

[2] Andriulli A, Loperfido S, Napolitano G, et al. Incidence rates of post-ERCP complications: a systematic survey of prospective studies. Am J Gastroenterol 2007;102:1781–8. 10.1111/j.1572-0241.2007.01279.x. 17509029

[3] Theopistos V, Theocharis G, Konstantakis C, et al. Non-operative Management of Type 2 ERCP-related retroperitoneal duodenal perforations: a 9-year experience from a single center. Gastroenterol Res 2018;11:207–12. 10.14740/gr1007w. PMC5997477 29915631

[4] Amini A, Lopez RA. Duodenal Perforation. In: StatPearls. StatPearls Publishing; 2024. Accessed September 30, 2024. http://www.ncbi.nlm.nih.gov/books/NBK553084/. 31971724

[5] Ansari D, Torén W, Lindberg S, et al. Diagnosis and management of duodenal perforations: a narrative review. Scand J Gastroenterol 2019;54:939–44. 10.1080/00365521.2019.1647456. 31353983

[6] Clinch D, Damaskos D, Di Marzo F, et al. Duodenal ulcer perforation: a systematic literature review and narrative description of surgical techniques used to treat large duodenal defects. J Trauma Acute Care Surg 2021;91:748–58. 10.1097/TA.0000000000003357. 34254960

[7] Kokkinakis S, Kritsotakis EI, Maliotis N, et al. Complications of modern pancreaticoduodenectomy: a systematic review and meta-analysis. Hepatobiliary Pancreat Dis Int 2022;21:527–37. 10.1016/j.hbpd.2022.04.006. 35513962

