# Correction to: A novel surgical technique for repairing duodenal and bile duct perforations following endoscopic retrograde cholangiopancreatography

**DOI:** 10.1093/jscr/rjag632

**Published:** 2026-07-16

This is a correction to: Hana Abdirahman, Omar Barakat, Alexis Nichols, Oluwatobi Soares, Jared Mortus, Vivi Chen, A novel surgical technique for repairing duodenal and bile duct perforations following endoscopic retrograde cholangiopancreatography, *Journal of Surgical Case Reports*, Volume 2025, Issue 2, February 2025, rjaf050, https://doi.org/10.1093/jscr/rjaf050

In the originally published version of this manuscript, the legend for Figure 3 was incomplete.

The legend should read: ``Schematic delineating the reconstruction technique for the vascularized, isolated gastric pouch. (A) Depiction of the site of the duodenal and bile duct injury. (B) Creation of the distal gastric pouch. (C) Final anastomoses of the pouch to the duodenal and bile duct defect. Copyright Baylor College of Medicine''.

Additionally, an acknowledgement was omitted from the published manuscript.

This should read: ``The authors would like to thank Scott Holmes, CMI, a member of the Michael E. DeBakey Department of Surgery at Baylor College of Medicine, for graphic assistance during the preparation of this manuscript''.

These details have been corrected only in this correction notice to preserve the version of record.

